# New bounds of the smoothing parameter for lattices

**DOI:** 10.1371/journal.pone.0328688

**Published:** 2025-07-24

**Authors:** Heng Guo, Fengxia Liu, Linlin Wang, Kun Tian

**Affiliations:** 1 School of Mathematics, Renmin University of China, Beijing, China; 2 School of Interdisciplinary Studies, Renmin University of China, Beijing, China; 3 Great Bay University, Dongguan, Guangdong, China; 4 Institute of Mathematics, Henan Academy of Sciences, Zhengzhou, Henan, China; Chinese Academy of Sciences Academy of Mathematics and Systems Science, CHINA

## Abstract

The smoothing parameter on lattices is crucial for lattice-based cryptographic design. In this study, we establish a new upper bound for the lattice smoothing parameter, which represents an improvement over several significant classical findings. For one-dimensional integer lattices, under specific and optimized conditions, we have achieved a more precise upper bound compared to previous research. Regarding general high-dimensional lattices, when the lattice dimension is large enough and the error parameter is within a particular range, we have derived a new upper bound. In the practical applications of lattice-based cryptography, where the lattice dimension is typically large, our new bound enables a more natural and smaller setting for the error parameter, thereby improving the upper bounds on all known smoothing parameters.

## 1 Introduction

In recent decades, the concept of a lattice has played a crucial role in post-quantum cryptography [1]. An *n*-dimensional (full-rank) lattice can be regarded as a discrete additive subgroup of ${\mathbb{R}}^{n}$ or a ℤ-module. Specifically, a lattice ℒ can be written as


$$ℒ=ℒ(𝐁)=\{\sum_{i=1}^{n}c_{i}{𝐛}_{i}|c_{i}\in \mathbb{Z}\},$$

where $𝐁=\{b_{1},b_{2},...,b_{n}\}\subseteq {\mathbb{R}}^{n}$ is a matrix composed of *n* linearly independent vectors, known as the generating matrix of lattice ℒ, and $\left\{b_{1},b_{2},...,b_{n}\right\}$ is called a basis for the lattice. The dual lattice ${ℒ}^{⋆}$ of a lattice ℒ is defined as:


$${ℒ}^{⋆}=\{𝐲\in {\mathbb{R}}^{n}|\langle 𝐱,𝐲\rangle \in \mathbb{Z},\forall 𝐱\in ℒ\}.$$

Lattice-based cryptography relies on the assumption that certain problems on lattices in ${\mathbb{R}}^{n}$ are hard, which serves as the cornerstone of secure cryptographic systems. Two of the most typical hard problems are the Shortest Vector Problem (SVP) and the Closest Vector Problem (CVP). It is currently believed that hard problems on lattices can effectively withstand quantum attacks. As a result, public-key cryptosystems based on lattice-based hard problems have become fundamental approaches and technologies in the field of post-quantum cryptography.

In lattice-based cryptography research, the discrete Gaussian measure defined on lattices is a highly important analytical tool. The discrete Gaussian measure was first used for this purpose by Regev and Micciancio [2,3] and was also developed on the basis of Banaszczyk’s proof of the transference theorems [4–6]. In [3], Micciancio and Regev defined an important concept related to the discrete Gaussian measure: the smoothing parameter $\eta_{\varepsilon }(ℒ)$. For any arbitrary *n*-dimensional lattice $ℒ\subseteq {\mathbb{R}}^{n}$ and any given ε>0, the smoothing parameter of lattice ℒ is defined as:


$$\eta_{\varepsilon }(ℒ)=min\{s|s>0,\rho_{1/s}({ℒ}^{⋆})\leq 1+\varepsilon \},$$

where $\rho_{1/s}$ is the discrete Gaussian distribution, which is defined formally in Definition 4. With the advancement of lattice cryptography, the smoothing parameter plays an increasingly crucial role in lattice sampling and hardness reductions (for details, see [3,7–10]). It can be argued that the concept of the smoothing parameter is equally important to the shortest vector, the successive minima of a lattice, and the covering radius.

In the study of smoothing parameters, one of the most important tasks is the estimation of bounds for the smoothing parameter. This is because, in lattice-based cryptography, improving the upper bound of the smoothing parameter generally enhances the security proofs of cryptographic systems and optimizes parameter settings, thereby improving the efficiency of cryptographic schemes (for details, see [3,7,8,10]). The originators of the smoothing parameter, Regev and Micciancio, first provided two estimation results in [3]. The first one is: for any lattice $ℒ\subseteq {\mathbb{R}}^{n}$,


$$\eta_{\varepsilon }(ℒ)\leq \sqrt{n}/\lambda_{1}({ℒ}^{⋆}),$$

where $\varepsilon =2^{-n}$. In [11] (see Chapter 1), Zheng and Tian provided two tighter results under the same conditions:


$$\eta_{\varepsilon }(ℒ)\leq r\sqrt{n}/\lambda_{1}({ℒ}^{⋆}),\eta_{\varepsilon }(ℒ)\leq \frac{4}{5}\sqrt{n}/\lambda_{1}({ℒ}^{⋆}),$$

where $r=\sqrt{\frac{1}{2\pi }+\frac{\ln2\pi }{2\pi }+\frac{1}{n\pi }\ln(1+2^{n})}(\leq 0.82)$. Regev and Micciancio provided the second non-trivial but very important conclusion in [3]:

$$\eta_{\varepsilon }(ℒ)\leq \sqrt{\frac{\ln(2n(1+\frac{1}{\varepsilon }))}{\pi }}\lambda_{n}(ℒ),$$ (1)

where $\lambda_{n}(ℒ)$ is the *n*-th successive minimum. Subsequently, in [12], Peikert combined the conclusion from Banaszczyk [5] to provide a tighter bound for $\eta_{\varepsilon }(ℒ)$ under the $l_{\infty }$-norm:

$$\eta_{\varepsilon }(ℒ)\leq \sqrt{\frac{\ln(2n(1+\frac{1}{\varepsilon }))}{\pi }}/\lambda_{1}^{\infty }({ℒ}^{⋆}).$$ (2)

Let $𝐁=\{b_{1},b_{2},...,b_{n}\}$ be a basis of ℒ, $\tilde{𝐁}=\{\tilde{{𝐛}_{1}},\tilde{{𝐛}_{2}},...,\tilde{{𝐛}_{n}}\}$ is the Gram-Schmidt orthogonalization of $\{b_{1},b_{2},...,b_{n}\}.$ Define $\tilde{bl}(ℒ)$ as :


$$\tilde{bl}(ℒ)=\min_{𝐁}\|\tilde{𝐁}\|=\min_{𝐁}\max_{1\leq i\leq n}\|{\tilde{𝐛}}_{i}\|.$$

In [8], Gentry, Peikert, and Vaikuntanathan established the result


$$1/\lambda_{1}^{\infty }({ℒ}^{⋆})\leq \tilde{bl}(ℒ)\leq \lambda_{n}(ℒ),$$

thus, they derived a new bound:

$$\eta_{\varepsilon }(ℒ)\leq \sqrt{\frac{\ln(2n(1+\frac{1}{\varepsilon }))}{\pi }}\tilde{bl}(ℒ).$$ (3)

Among (1), (2), and (3), it is evident that (2) provides the best estimate.

In recent research [13], Zhongxiang Zheng, Guangwu Xu, and Chunhuan Zhao derived an estimate of the smoothing parameter for the one-dimensional integer lattice ℤ:

$$\eta_{\varepsilon }(\mathbb{Z})\leq \sqrt{\frac{\ln(\frac{\varepsilon }{44}+\frac{2}{\varepsilon })}{\pi }},$$ (4)

with 0<ε<0.086435. Next, they established a relationship between the one-dimensional integer lattice ℤ and a general lattice $ℒ\subseteq {\mathbb{R}}^{n}$, achieving significant improvements when 0<ε<min{1,0.086435n}:

$$\eta_{\varepsilon }(ℒ)\leq \sqrt{\frac{\ln(n-1+\frac{2n}{\varepsilon })}{\pi }}\tilde{bl}(ℒ).$$ (5)

In other works [10,14–17], Kai-Min Chung *et al*. approximated the complexity of the smoothing parameter within a constant factor [15]. Thomas Espitau *et al*. [10], Wei *et al*. [14], Elena Kirshanova *et al*. [16], and Zheng Zhiyong *et al*. [17] provided corresponding bounds for the smoothing parameter for certain special lattices. Each of these results has had a significant impact in its respective article.

**Contributions.** In this paper, we obtain a new upper bound. First, for the one-dimensional integer lattice ℤ, when $0<\varepsilon \leq \frac{4}{m}-\frac{172}{m^{6}}$, where m≥3 , we have:


$$\eta_{\varepsilon }(\mathbb{Z})\leq \sqrt{\frac{\ln(\frac{\varepsilon }{m}+\frac{2}{\varepsilon })}{\pi }}.$$

Secondly, for a general *n*-dimensional lattice ℒ, when *n* is sufficiently large such that $\sqrt{\frac{24n+1}{n^{2}-1}}\leq \varepsilon \leq \sqrt{2}-1$, we have:


$$\eta_{\varepsilon }(ℒ)\leq \sqrt{\frac{\ln((n-1)(1+\frac{2}{\varepsilon }))}{\pi }}\tilde{bl}(ℒ).$$

Our results outperform those in [8] and [13], and a detailed discussion will be presented in the section entitled "Comparative Analysis of Smoothness Parameter Bounds".

It is worth noting that, in our proof process, we can derive the following relationship between a general *n*-dimensional lattice ℒ and the *n*-dimensional integer lattice ${\mathbb{Z}}^{n}$:


$$\eta_{\varepsilon }(ℒ)\leq \tilde{bl}(ℒ)\eta_{\varepsilon }({\mathbb{Z}}^{n}).$$

Furthermore, we can prove that for the smoothing parameter $\eta_{\varepsilon }({\mathbb{Z}}^{n})$ of the *n*-dimensional integer lattice ${\mathbb{Z}}^{n}$, under the same conditions,


$$\eta_{\varepsilon }({\mathbb{Z}}^{n})\leq \sqrt{\frac{\ln((n-1)(1+\frac{2}{\varepsilon }))}{\pi }}.$$

**Organization.** The rest of the paper is organized as follows. In the second section, we introduce the necessary basic concepts and preliminary knowledge. In Sect 3, we present the new upper bounds for the smoothing parameter $\eta_{\varepsilon }(\mathbb{Z})$ of the one-dimensional integer lattice, $\eta_{\varepsilon }({\mathbb{Z}}^{n})$ of the *n*-dimensional integer lattice, and $\eta_{\varepsilon }(ℒ)$ of the general *n*-dimensional lattice. Sect 4 presents a comparison of numerical results. The final part is a summary.

## 2 Preliminaries

### 2.1 General notation

The bold capital ℤ represents the integer ring. The symbol ${\mathbb{R}}^{n}$ denotes the *n*-dimensional real linear space. Lowercase letters in bold, such as 𝐱,𝐲,𝐚 and **b**, represent vectors in ${\mathbb{R}}^{n}$. The standard inner product in ${\mathbb{R}}^{n}$ is denoted by ⟨,⟩. The Euclidean norm of a vector $𝐱=(x_{1},x_{2},...,x_{n})\in {\mathbb{R}}^{n}$ is


$$|𝐱|=∥𝐱{∥}_{2}=\sqrt{\sum_{i=1}^{n}x_{i}^{2}}.$$

For any $𝐒\in {\mathbb{R}}^{n\times m}$, if ${𝐬}_{1},\ldots ,{𝐬}_{m}$ denote its columns, then we define


$$\|𝐒\|=\max_{1\leq i\leq m}|{𝐬}_{i}|.$$

Let N=N(0,1) denote the unit ball in ${\mathbb{R}}^{n}$, that is $N=N(0,1)=\{𝐱\in {\mathbb{R}}^{n}|\;|𝐱|<1\}$. A lattice ℒ is a discrete geometry in ${\mathbb{R}}^{n}$, in other words, there is a positive constant $\lambda_{1}=\lambda_{1}(ℒ)>0$, and a nonzero vector 𝐚∈ℒ such that


$$|𝐚|=\min_{𝐱\in ℒ,𝐱\neq 0}|𝐱|=\lambda_{1}(ℒ).$$

We call $\lambda_{i}(ℒ)(1\leq i\leq n)$ the ith successive minimum distance of lattice ℒ if


$$\lambda_{i}(ℒ)=min\{r|dim(span(ℒ\cap rN(0,1)))\geq i\}.$$

For any lattice ℒ(𝐁) with basis matrix **B** whose columns are ${𝐛}_{1},\ldots ,{𝐛}_{n}$, the set


$$F(𝐁)=\left\{\sum_{i=1}^{n}x_{i}{𝐛}_{i}\;|\;x_{i}\in [0,1),\;1\leq i\leq n\right\}$$

is the basic neighborhood of the lattice ℒ(𝐁). Therefore, a basic neighborhood F(𝐁) of a lattice ℒ(𝐁) is the set of representative elements of the additive quotient group ${\mathbb{R}}^{n}/ℒ(𝐁)$, and any basic neighborhood F(𝐁) of an arbitrary lattice ℒ(𝐁) forms an additive group mod ℒ(𝐁). Let the determinant of the lattice be d(ℒ) and the volume of basic neighborhood be Vol(F(𝐁)), then d(ℒ)=Vol(F(𝐁)). If *f*(*x*) is a function defined on ${\mathbb{R}}^{n}$ and $A\subseteq {\mathbb{R}}^{n}$ is a countable set, denote $f(A)=\sum_{𝐱\in A}f(𝐱).$

### 2.2 Discrete Gaussian measure and related conclusions

In this subsection, we will directly provide the definition and related properties of the discrete Gaussian measure, which will play an important role in the third section.

**Definition 1.** *Let s>0 be a given positive real number, $𝐜\in {\mathbb{R}}^{n}$ be a given vector. With* **c** *as the center, the Gaussian function $\rho_{s,𝐜}(𝐱)$ with parameter s is defined as:*


$$\rho_{s,𝐜}(𝐱)=e^{-\frac{\pi }{s^{2}}|𝐱-𝐜{|}^{2}},𝐱\in {\mathbb{R}}^{n}.$$

**Definition 2.** *If $f(𝐱)\in L^{1}({\mathbb{R}}^{n})$, the Fourier transform $\hat{f}(𝐱)$ of f(𝐱) is defined as:*


$$\hat{f}(𝐱)=\int_{{\mathbb{R}}^{n}}f(𝐲)e^{-2\pi i\langle 𝐱,𝐲\rangle }d𝐲.$$

The following are some of the most commonly used and basic properties of the Fourier transform [11].

**Lemma 3.** *If $f(𝐱)\in L^{1}({\mathbb{R}}^{n})$, $g(𝐱)\in L^{1}({\mathbb{R}}^{n})$, and $f*g(𝐱)=\int_{{\mathbb{R}}^{n}}f(𝐱-\xi )g(\xi )\;d\xi$, then*

(1) $\hat{f*g}(𝐱)=\hat{f}(𝐱)\hat{g}(𝐱);$

(2) *If $h(𝐱)=e^{2\pi i\langle 𝐱,𝐚\rangle }f(𝐱)$, then $\hat{h}(𝐱)=\hat{f}(x-a);$*

(3) *If s≠0, $f_{s}(𝐱)=f\left(\frac{1}{s}𝐱\right)$, then $\hat{f_{s}}(𝐱)=|s{|}^{n}{\hat{f}}_{1/s}(𝐱)=|s{|}^{n}\hat{f}(s𝐱).$*

Therefore, the Fourier transform of the Gaussian functions $\rho_{s}(𝐱)$ and $\rho_{s,𝐜}(𝐱)$ is: ${\hat{\rho }}_{s}(𝐱)=s^{n}\rho_{1/s}(𝐱)=s^{n}e^{-\pi |s𝐱{|}^{2}}$ and ${\hat{\rho }}_{s,𝐜}(𝐱)=e^{-2\pi i\langle 𝐱,𝐜\rangle }s^{n}\rho_{1/s}(𝐱)$, respectively.

**Definition 4.** *Let 𝐱∈ℒ and s>0 be a given positive real number, $𝐜\in {\mathbb{R}}^{n}$ be a given vector. The discrete Gaussian measure $D_{ℒ,s,𝐜}(𝐱)$ defined on lattice ℒ is:*


$$D_{ℒ,s,𝐜}(𝐱)=\frac{\rho_{s,𝐜}(𝐱)}{\rho_{s,𝐜}(ℒ)}.$$

In other words, $D_{ℒ,s,𝐜}(𝐱)$ represents the probability of a single lattice point. Below we provide two very useful lemmas, which are proved in detail in [11].

**Lemma 5.** *If $f(𝐱)\in L^{1}({\mathbb{R}}^{n})$, and the following two conditions are satisfied:*

(1) $\sum_{𝐱\in ℒ}|f(x+c)|$* converges uniformly with respect to* **c** *in any closed region of ${\mathbb{R}}^{n}$;*

(2) *Assume that the series $\sum_{𝐲\in {ℒ}^{⋆}}|\hat{f}(𝐲)|$ converges. Then we have*


$$\sum_{𝐱\in ℒ}f(𝐱)=\frac{1}{d(ℒ)}\sum_{𝐲\in {ℒ}^{⋆}}\hat{f}(𝐲).$$

**Lemma 6.** *For any n-dimensional lattice ℒ, s>0, $𝐜\in {\mathbb{R}}^{n}$, there is $\rho_{s,𝐜}(ℒ)\leq \rho_{s}(ℒ)$.*

## 3 New upper bound on smoothing parameter

In this section, we will prove the new upper bound on smoothing parameters. Following [13], we begin with the one-dimensional integer lattice ℤ and then extend to the general lattice ℒ.

### 3.1 New upper bound on the smoothing parameter for the integer lattice ℤ

In [13], Zheng *et al*. provide the relationship between $\eta_{\varepsilon }(ℒ)$ for general lattices and $\eta_{\varepsilon }(\mathbb{Z})$ for the one-dimensional integer lattice ℤ, emphasizing the importance of determining an upper bound for $\eta_{\varepsilon }(\mathbb{Z})$. Additionally, in lattice sampling, the one-dimensional integer lattice ℤ serves as a basis for sampling higher-dimensional lattices, as detailed in [10,18–22]. Therefore, achieving a more accurate computation of $\eta_{\varepsilon }(\mathbb{Z})$ will be of considerable importance.

Let’s start with a few lemmas.

**Lemma 7.** *If 0<ε<1, then $\eta_{\varepsilon }(\mathbb{Z})>\sqrt{\frac{\ln(\frac{2}{\varepsilon })}{\pi }}$. Furthermore, if $\rho_{1/s}(\mathbb{Z})<1\;+\;\varepsilon$ , then $\pi s^{2}>\ln2$.*

*Proof*: Since ℤ is a self-dual lattice, according to the definition of the smoothing parameter, $\eta_{\varepsilon }(\mathbb{Z})=min\{s|s>0,\rho_{1/s}(\mathbb{Z})\leq 1+\varepsilon \}$. If


$$\rho_{1/s}(\mathbb{Z})=\sum_{j\in \mathbb{Z}}e^{-\pi s^{2}j^{2}}=1+2\sum_{j=1}^{+\infty }e^{-\pi s^{2}j^{2}}>1+2e^{-\pi s^{2}}>1+\varepsilon ,$$

then, $s\leq \sqrt{\frac{\ln(\frac{2}{\varepsilon })}{\pi }}$, which implies $\eta_{\varepsilon }(\mathbb{Z})>\sqrt{\frac{\ln(\frac{2}{\varepsilon })}{\pi }}$. Furthermore, if $\rho_{1/s}(\mathbb{Z})\leq 1+\varepsilon$, then $s>\sqrt{\frac{\ln(\frac{2}{\varepsilon })}{\pi }}$, which means $e^{\pi s^{2}}>\frac{2}{\varepsilon }>2$, and thus $\pi s^{2}>\ln2$. ◻

**Lemma 8.** *If x>0, then $e^{-x}\leq \frac{1}{ex}.$*

*Proof*: To prove that $e^{-x}\leq \frac{1}{ex}$ for *x*>0 is equivalent to proving that $e^{x}\geq ex$. We define the function *f*(*x*) = *e*^*x*^−*ex* for *x*>0. Taking the derivative of *f*(*x*), we get:


$$f^{\prime }(x)=e^{x}-e.$$

Thus, when *x*>1, $f^{\prime }(x)>0$, and when 0<x≤1, $f^{\prime }(x)\leq 0$. This shows that *f*(*x*) is decreasing for 0<x≤1 and increasing for *x*>1, which implies that *f*(*x*) attains its minimum value at *x* = 1. Therefore, f(x)≥f(1)=0, which leads to the conclusion that for *x*>0, $e^{x}\geq ex$. ◻

**Lemma 9.** *If 0<ε<1, and $s>\sqrt{\frac{\ln(\frac{2}{\varepsilon })}{\pi }}$, then*

$$\sum_{j=1}^{+\infty }e^{-\pi s^{2}j^{2}}<e^{-\pi s^{2}}+e^{-4\pi s^{2}}+\frac{4}{3}e^{-9\pi s^{2}}.$$ (6)

*Proof*: To prove (6), it is equivalent to proving


$$\sum_{i=4}^{+\infty }e^{-\pi s^{2}j^{2}}=e^{-16\pi s^{2}}+e^{-25\pi s^{2}}+e^{-36\pi s^{2}}...<\frac{1}{3}e^{-9\pi s^{2}},$$

which means proving:


$$e^{-(4^{2}-3^{2})\pi s^{2}}+e^{-(5^{2}-3^{2})\pi s^{2}}+e^{-(6^{2}-3^{2})\pi s^{2}}...<\frac{1}{3}.$$

According to Lemma 8, when *x* > 0, it holds that $e^{-x}\leq \frac{1}{ex}$. Therefore,


$$e^{-(4^{2}-3^{2})\pi s^{2}}+e^{-(5^{2}-3^{2})\pi s^{2}}+e^{-(6^{2}-3^{2})\pi s^{2}}+...$$

$$\leq \frac{1}{e\pi s^{2}}(\frac{1}{4^{2}-3^{2}}+\frac{1}{5^{2}-3^{2}}+...+\frac{1}{n^{2}-3^{2}}+...)$$

$$\leq \frac{1}{e\pi s^{2}}(\frac{1}{7}+\frac{1}{4^{2}}+\frac{1}{5^{2}}+...+\frac{1}{(n-1{)}^{2}}+...)$$

$$\leq \frac{1}{e\pi s^{2}}(\frac{1}{7}+\frac{1}{3\times 4}+\frac{1}{4\times 5}+...+\frac{1}{\left(n-2)\times (n-1\right)}+...)$$

$$=\frac{1}{e\pi s^{2}}(\frac{1}{7}+(\frac{1}{3}-\frac{1}{4})+(\frac{1}{4}-\frac{1}{5})+...+(\frac{1}{n-2}-\frac{1}{n-1})+...)$$

$$=\frac{1}{e\pi s^{2}}(\frac{1}{7}+\frac{1}{3})$$

$$\leq \frac{10}{21e\pi s^{2}}$$

$$<\frac{1}{3}.$$

The last equality holds because, by the assumption $s>\sqrt{\frac{\ln(\frac{2}{\varepsilon })}{\pi }}$, we have $\pi s^{2}>\ln2$, which implies $21e\pi s^{2}>30$. This completes the proof. ◻

We will now directly prove our result. Compared to the method in [13], our proof is more optimized and concise.

**Theorem 10.** *Let m≥3. When $0<\varepsilon \leq \frac{4}{m}-\frac{172}{m^{6}}$ , we have:*


$$\eta_{\varepsilon }(\mathbb{Z})\leq \sqrt{\frac{\ln(\frac{\varepsilon }{m}+\frac{2}{\varepsilon })}{\pi }}.$$

*Proof*: Let $\alpha =\frac{m\varepsilon }{2m+\varepsilon^{2}}$. It is evident that α<1. When $s\geq \sqrt{\frac{\ln(\frac{\varepsilon }{m}+\frac{2}{\varepsilon })}{\pi }}$, we have $e^{-\pi s^{2}}\leq \frac{m\varepsilon }{2m+\varepsilon^{2}}$. Let $X=e^{-\pi s^{2}}$. We will now prove that

$$X+X^{4}+\frac{4}{3}X^{9}\leq \alpha +\alpha^{4}+\frac{4}{3}\alpha^{9}<\frac{\varepsilon }{2}.$$ (7)

Substitute $\alpha =\frac{m\varepsilon }{2m+\varepsilon^{2}}$ into the expression, and let $\varepsilon =\frac{4}{m}-\delta$, where $\frac{4}{m}>\delta >\frac{172}{m^{6}}$. Then,


$$\frac{m\varepsilon }{2m+\varepsilon^{2}}+\frac{m^{4}\varepsilon^{4}}{(2m+\varepsilon^{2}{)}^{4}}+\frac{4}{3}\frac{m^{9}\varepsilon^{9}}{(2m+\varepsilon^{2}{)}^{9}}<\frac{\varepsilon }{2}$$

$$\Leftrightarrow \frac{m^{4}\varepsilon^{4}}{(2m+\varepsilon^{2}{)}^{4}}+\frac{4}{3}\frac{m^{9}\varepsilon^{9}}{(2m+\varepsilon^{2}{)}^{9}}<\frac{\varepsilon^{3}}{2(2m+\varepsilon^{2})}$$

$$\Leftrightarrow \frac{8}{3}m^{9}\varepsilon^{6}+2m^{4}\varepsilon (2m+\varepsilon^{2}{)}^{5}<(2m+\varepsilon^{2}{)}^{8}$$

$$\Leftrightarrow \frac{8}{3}m^{9}\varepsilon^{6}<(2m+\varepsilon^{2}{)}^{5}((2m+\varepsilon^{2}{)}^{3}-2m^{4}\varepsilon )$$

$$\Leftrightarrow \frac{8}{3}m^{9}\varepsilon^{6}<(2m+\varepsilon^{2}{)}^{5}((2m+\varepsilon^{2}{)}^{3}-2m^{4}(\frac{4}{m}-\delta ))$$

$$\Leftrightarrow \frac{8}{3}m^{9}\varepsilon^{6}<(2m+\varepsilon^{2}{)}^{5}(12m^{2}\varepsilon^{2}+6m\varepsilon^{4}+2m^{4}\delta +\varepsilon^{6}).$$

When $\varepsilon \leq \frac{4}{m}-\frac{172}{m^{6}}$, we have


$$\varepsilon <\frac{4}{m}\Rightarrow \varepsilon^{6}<\frac{4096}{m^{6}}\Rightarrow \frac{8}{3}\varepsilon^{6}<\frac{8}{3}\cdot \frac{4096}{m^{6}}<64\cdot \frac{172}{m^{6}}<64\delta .$$

Therefore,


$$\frac{8}{3}m^{9}\varepsilon^{6}<64m^{9}\delta <(2m+\varepsilon^{2}{)}^{5}(12m^{2}\varepsilon^{2}+6m\varepsilon^{4}+2m^{4}\delta +\varepsilon^{6}).$$

Note that the coefficient of the $m^{9}\delta$ term on the right side of the inequality is exactly 64. Therefore, (7) holds.

According to Lemma 9, when $s\geq \sqrt{\frac{\ln(\frac{\varepsilon }{m}+\frac{2}{\varepsilon })}{\pi }}$, it follows that


$$\rho_{\frac{1}{s}}(\mathbb{Z})=1+2\left(e^{-\pi s^{2}}+e^{-4\pi s^{2}}+e^{-9\pi s^{2}}+e^{-16\pi s^{2}}+e^{-25\pi s^{2}}+\cdots \right)$$

$$<1+2\left(e^{-\pi s^{2}}+e^{-4\pi s^{2}}+\frac{4}{3}e^{-9\pi s^{2}}\right)$$

$$=1+2\left(X+X^{4}+\frac{4}{3}X^{9}\right)$$

$$\leq 1+2\left(\alpha +\alpha^{4}+\frac{4}{3}\alpha^{9}\right)\leq 1+\varepsilon$$

Therefore,


$$\eta_{\varepsilon }(\mathbb{Z})\leq \sqrt{\frac{\ln(\frac{\varepsilon }{m}+\frac{2}{\varepsilon })}{\pi }}.$$

Proof complete. ◻

**Remark 1.**

(1) Compared to (4), our conclusions improve for the same choice of ε when *m*>44.

(2) Additionally, since *m* can vary, our results also have a broader applicability in the estimation of one-dimensional integer lattice smoothing parameters.

As an application of Theorem 10, we derive a new upper bound for the smoothing parameter of a general one-dimensional lattice aℤ.

**Corollary 11.** *Let m≥3, and $0<\varepsilon \leq \frac{4}{m}\;-\;\frac{172}{m^{6}}$. For a∈ℝ with a≠0, then for any one-dimensional lattice aℤ, we have*


$$\eta_{\varepsilon }(a\mathbb{Z})\leq |a|\sqrt{\frac{\ln(\frac{\varepsilon }{m}+\frac{2}{\varepsilon })}{\pi }}$$

*Proof*: The dual lattice of a one-dimensional lattice aℤ is $\frac{1}{a}\mathbb{Z}$. By the definition of the smoothing parameter,


$$\eta_{\varepsilon }(a\mathbb{Z})=\min\{s|s>0,\rho_{1/s}(\frac{1}{a}\mathbb{Z})\leq 1+\varepsilon \}=\min\{s|s>0,\rho_{|a|/s}(\mathbb{Z})\leq 1+\varepsilon \}.$$

By following the proof process of Theorem 10, we obtain


$$\eta_{\varepsilon }(a\mathbb{Z})\leq |a|\sqrt{\frac{\ln(\frac{\varepsilon }{m}+\frac{2}{\varepsilon })}{\pi }}.*\mathrm{-1pc}$$

◻

At the end of this subsection, we note that the formal upper bound of the smoothing parameter $\eta_{\varepsilon }({\mathbb{Z}}^{n})$ for the *n*-dimensional integer lattice ${\mathbb{Z}}^{n}$ can be derived from Theorem 10, and which provides a basis for finding a more precise upper bound for $\eta_{\varepsilon }({\mathbb{Z}}^{n})$ in the next section.

**Corollary 12.** *Let 0<ε≤1 , then:*


$$\eta_{\varepsilon }({\mathbb{Z}}^{n})\leq \sqrt{\frac{\ln(\frac{\varepsilon^{\prime }}{3}+\frac{2}{\varepsilon^{\prime }})}{\pi }},$$

*where $\varepsilon^{\prime }=\sqrt[{n}]{1+\varepsilon }-1.$*

*Proof*: In Theorem 10, by setting *m* = 3, and for 0<ε≤1, we have


$$\eta_{\varepsilon }(\mathbb{Z})\leq \sqrt{\frac{\ln(\frac{\varepsilon }{3}+\frac{2}{\varepsilon })}{\pi }}.$$

Note that ${\mathbb{Z}}^{n}$ is also self-dual, and the series appearing in the following equations are all absolutely convergent. Therefore,


$$\eta_{\varepsilon }({\mathbb{Z}}^{n})=min\{s|s>0,\sum_{𝐱=(x_{1},x_{2},...,x_{n})\in {\mathbb{Z}}^{n}}e^{-\pi s^{2}|𝐱{|}^{2}}\leq 1+\varepsilon \}$$

$$=min\{s|s>0,\sum_{x_{1},x_{2},...,x_{n}\in \mathbb{Z}}e^{-\pi s^{2}\sum_{i=1}^{n}x_{i}^{2}}\leq 1+\varepsilon \}$$

$$=min\{s|s>0,\prod_{i=1}^{n}\sum_{x_{i}\in \mathbb{Z}}e^{-\pi s^{2}|x_{i}{|}^{2}}\leq 1+\varepsilon \}$$

$$=min\{s|s>0,(\sum_{x\in \mathbb{Z}}e^{-\pi s^{2}|x{|}^{2}}{)}^{n}\leq 1+\varepsilon \}$$

$$=min\{s|s>0,\rho_{1/s}(\mathbb{Z})\leq \sqrt[{n}]{1+\varepsilon }\}$$

$$=min\{s|s>0,\rho_{1/s}(\mathbb{Z})\leq 1+(\sqrt[{n}]{1+\varepsilon }-1)\}.$$

Let $\varepsilon^{\prime }=\sqrt[{n}]{1+\varepsilon }-1,$ then $\eta_{\varepsilon }({\mathbb{Z}}^{n})=\eta_{\varepsilon^{\prime }}(\mathbb{Z})$. When 0<ε<1 , then $(1+\varepsilon )<(1+\varepsilon {)}^{n},\varepsilon^{\prime }=\sqrt[{n}]{1+\varepsilon }-1<\varepsilon <1$, Therefore


$$\eta_{\varepsilon }({\mathbb{Z}}^{n})\leq \sqrt{\frac{\ln(\frac{\varepsilon^{\prime }}{3}+\frac{2}{\varepsilon^{\prime }})}{\pi }}.$$

◻

### 3.2 The new upper bound on the smoothing parameter for the *n*-dimensional integer lattice ${\mathbb{Z}}^{n}$ and general lattice ℒ

This subsection mainly presents two results. Firstly, it provides a new upper bound for the smoothing parameter of the *n*-dimensional integer lattice ${\mathbb{Z}}^{n}$ (Theorem 15). Secondly, we obtain that $\eta_{\varepsilon }({\mathbb{Z}}^{n})$ and $\eta_{\varepsilon }(ℒ)$ satisfy a certain inequality (Lemma 16), which allows us to derive a new upper bound for the smoothing parameter $\eta_{\varepsilon }(ℒ)$ of a general lattice ℒ (Theorem 17).

For readability, we will reintroduce some of the notation.

Let $𝐁=\{b_{1},b_{2},...,b_{n}\}$ be a basis for the lattice ℒ . $\tilde{𝐁}=\{\tilde{{𝐛}_{1}},\tilde{{𝐛}_{2}},...,\tilde{{𝐛}_{𝐧}}\}$ represents the Gram-Schmidt orthogonalization of this basis, i.e.,


$$\tilde{{𝐛}_{1}}=b_{1},\;\tilde{{𝐛}_{𝐢}}=b_{i}-\sum_{j=1}^{i-1}\frac{\langle b_{i},\tilde{{𝐛}_{𝐣}}\rangle }{\langle \tilde{{𝐛}_{𝐣}},\tilde{{𝐛}_{𝐣}}\rangle }\tilde{{𝐛}_{𝐣}},\;2\leq i\leq n.$$

Then, there exists an upper triangular matrix


$$𝐔=(u_{i,j}{)}_{1\leq i,j\leq n}\in {\mathbb{R}}^{n\times n},u_{ii}=1,1\leq i\leq n,$$

such that $𝐁=\tilde{𝐁}𝐔.$

Define $\tilde{bl}(ℒ)$ as


$$\tilde{bl}(ℒ)=\min_{𝐁}\|\tilde{𝐁}\|=\min_{𝐁}\max_{1\leq i\leq n}\|{\tilde{𝐛}}_{i}\|.$$

Before proving the result, let us first present some necessary lemmas.

**Lemma 13.** *If $0\leq x\leq \sqrt{2}-1$, k is a positive integer, and 1≤k≤n−1, then*


$$(1+x{)}^{\frac{k}{n}}\leq 1+\frac{k}{n}x-\frac{k(n-k)}{4n^{2}}x^{2}.$$

*Proof*: Let $f(x)=(1+x{)}^{\frac{k}{n}}-1-\frac{k}{n}x+\frac{k(n-k)}{4n^{2}}x^{2}$, $0\leq x\leq \sqrt{2}-1.$ Then,


$$f^{\;\prime }(x)=\frac{k}{n}(1+x{)}^{\frac{k}{n}-1}-\frac{k}{n}+\frac{k(n-k)}{2n^{2}}x,$$

$$f^{\;\prime \prime }(x)=\frac{k}{n}(\frac{k}{n}-1)(1+x{)}^{\frac{k}{n}-2}+\frac{k(n-k)}{2n^{2}}.$$

Since $0\leq x\leq \sqrt{2}-1$, then $1\leq 1+x\leq \sqrt{2}$, hence $1\leq (1+x{)}^{2-\frac{k}{n}}\leq (1+x{)}^{2}\leq 2$, thus $\frac{1}{2}\leq (1+x{)}^{\frac{k}{n}-2}\leq 1$ .

Note that $\frac{k}{n}-1<0$, thus,


$$f^{\prime \prime }(x)\leq \frac{1}{2}\frac{k}{n}(\frac{k}{n}-1)+\frac{k(n-k)}{2n^{2}}=0.$$

So, $f^{\;\prime }(x)$ is monotonically decreasing. Furthermore, since $f^{\;\prime }(0)=0$, it follows that $f^{\;\prime }(x)\leq 0$, thus *f*(*x*) is also monotonically decreasing. Since f(0)=0, it follows that $f(x)\leq 0,0\leq x\leq \sqrt{2}-1$. That is, we have:


$$(1+x{)}^{\frac{k}{n}}\leq 1+\frac{k}{n}x-\frac{k(n-k)}{4n^{2}}x^{2}.$$

◻

**Lemma 14.** *If $0\leq \varepsilon \leq \sqrt{2}-1$, n is a positive integer, then*


$$\sqrt[{n}]{1+\varepsilon }-1\geq \frac{\varepsilon }{(1+\frac{\varepsilon }{2})n-\frac{\varepsilon }{2}-\frac{n^{2}-1}{24n}\varepsilon^{2}}.$$

*Proof*: Please note that the following equation holds true:


$$\sum_{k=1}^{n-1}k(n-k)=\sum_{k=1}^{n-1}nk-\sum_{k=1}^{n-1}k^{2}$$

$$=\frac{1}{2}(n-1)n^{2}-\frac{1}{6}(n-1)n(2n-1)$$

$$=\frac{1}{6}(n-1)n(n+1).$$

Therefore, combining the equation $x^{n}-1=(x-1)(x^{n-1}+...+x+1)$ and Lemma 13, we have:


$$\sqrt[{n}]{1+\varepsilon }-1=\frac{(\sqrt[{n}]{1+\varepsilon }{)}^{n}-1}{1+(1+\varepsilon {)}^{\frac{1}{n}}+(1+\varepsilon {)}^{\frac{2}{n}}+...+(1+\varepsilon {)}^{\frac{n-1}{n}}}$$

$$\geq \frac{\varepsilon }{1+[1+\frac{1}{n}\varepsilon -\frac{1\cdot (n-1)}{4n^{2}}\varepsilon^{2}]+...+[1+\frac{n-1}{n}\varepsilon -\frac{(n-1)\cdot 1}{4n^{2}}\varepsilon^{2}]}$$

$$=\frac{\varepsilon }{n+(\frac{1}{n}+\frac{2}{n}+...+\frac{n-1}{n})\varepsilon -\frac{\varepsilon^{2}}{4n^{2}}\sum_{k=1}^{n-1}k(n-k)}$$

$$=\frac{\varepsilon }{n+\frac{1}{n}\frac{n(n-1)}{2}\varepsilon -\frac{\varepsilon^{2}}{4n^{2}}\frac{1}{6}(n-1)n(n+1)}$$

$$=\frac{\varepsilon }{(1+\frac{\varepsilon }{2})n-\frac{\varepsilon }{2}-\frac{n^{2}-1}{24n}\varepsilon^{2}}.$$

◻

Next, we can provide a more precise upper bound for the smoothing parameter of the *n*-dimensional integer lattice ${\mathbb{Z}}^{n}$.

**Theorem 15.** *When n is sufficiently large such that $\sqrt{\frac{24n+1}{n^{2}-1}}\leq \varepsilon \leq \sqrt{2}-1$ , then*


$$\eta_{\varepsilon }({\mathbb{Z}}^{n})\leq \sqrt{\frac{\ln((n-1)(1+\frac{2}{\varepsilon }))}{\pi }}.$$

*Proof*: In Theorem 10, let *m* = 8. Then, when $0<\varepsilon^{\prime }\leq \sqrt{2}-1$, we have


$$\eta_{\varepsilon^{\prime }}(\mathbb{Z})\leq \sqrt{\frac{\ln(\frac{\varepsilon^{\prime }}{8}+\frac{2}{\varepsilon^{\prime }})}{\pi }}.$$

According to the definition of the smoothing parameter, then


$$\eta_{\varepsilon }({\mathbb{Z}}^{n})=min\{s|s>0,\sum_{𝐱=(x_{1},x_{2},...,x_{n})\in {\mathbb{Z}}^{n}}e^{-\pi s^{2}|𝐱{|}^{2}}\leq 1+\varepsilon \}$$

$$=min\{s|s>0,\sum_{x_{1},x_{2},...,x_{n}\in \mathbb{Z}}e^{-\pi s^{2}\sum_{i=1}^{n}x_{i}^{2}}\leq 1+\varepsilon \}$$

$$=min\{s|s>0,\prod_{i=1}^{n}\sum_{x_{i}\in \mathbb{Z}}e^{-\pi s^{2}|x_{i}{|}^{2}}\leq 1+\varepsilon \}$$

$$=min\{s|s>0,(\sum_{x\in \mathbb{Z}}e^{-\pi s^{2}|x{|}^{2}}{)}^{n}\leq 1+\varepsilon \}$$

$$=min\{s|s>0,(\rho_{1/s}(\mathbb{Z}){)}^{n}\leq 1+\varepsilon \}.$$

Let $\varepsilon^{\prime }=\frac{\varepsilon }{(1+\frac{\varepsilon }{2})n-\frac{\varepsilon }{2}-\frac{n^{2}-1}{24n}\varepsilon^{2}}$. Then, according to Lemma 14, we have $(1+\varepsilon^{\prime }{)}^{n}\leq 1+\varepsilon$. Next, we will prove that when $\sqrt{\frac{24n+1}{n^{2}-1}}\leq \varepsilon \leq \sqrt{2}-1$, we have $(n-1)(1+\frac{2}{\varepsilon })>\frac{\varepsilon^{\prime }}{8}+\frac{2}{\varepsilon^{\prime }}$.


$$\left(n-1)(1+\frac{2}{\varepsilon })-(\frac{\varepsilon^{\prime }}{8}+\frac{2}{\varepsilon^{\prime }}\right)$$

$$=(n-1)(1+\frac{2}{\varepsilon })-\frac{\varepsilon }{8((1+\frac{\varepsilon }{2})n-\frac{\varepsilon }{2}-\frac{n^{2}-1}{24n}\varepsilon^{2})}-\frac{2}{\frac{\varepsilon }{(1+\frac{\varepsilon }{2})n-\frac{\varepsilon }{2}-\frac{n^{2}-1}{24n}\varepsilon^{2}}}$$

$$=(n-1)(1+\frac{2}{\varepsilon })-(n-1+\frac{2n}{\varepsilon }-\frac{n^{2}-1}{12n}\varepsilon )-\frac{\varepsilon }{(8+4\varepsilon )n-4\varepsilon -\frac{n^{2}-1}{3n}\varepsilon^{2}}$$

$$>\frac{n^{2}-1}{12n}\varepsilon -\frac{2}{\varepsilon }-\frac{\varepsilon }{8n-4\varepsilon -\frac{n\varepsilon^{2}}{3}}$$

$$>\frac{n^{2}-1}{12n}\varepsilon -\frac{2}{\varepsilon }-\frac{\varepsilon }{6n}$$

$$=\frac{(n^{2}-1)\varepsilon^{2}-24n-2\varepsilon^{2}}{12n\varepsilon }$$

$$>\frac{(n^{2}-1)\varepsilon^{2}-24n-1}{12n\varepsilon }>0.$$

Therefore, if


$$s\geq \sqrt{\frac{\ln((n-1)(1+\frac{2}{\varepsilon }))}{\pi }}>\sqrt{\frac{\ln(\frac{\varepsilon^{\prime }}{8}+\frac{2}{\varepsilon^{\prime }})}{\pi }},$$

then


$$(\rho_{1/s}(\mathbb{Z}){)}^{n}\leq (1+\varepsilon^{\prime }{)}^{n}\leq 1+\varepsilon .$$

Therefore,


$$\eta_{\varepsilon }({\mathbb{Z}}^{n})\leq \sqrt{\frac{\ln((n-1)(1+\frac{2}{\varepsilon }))}{\pi }}.$$

◻

The following lemma shows the relationship between the smoothing parameter $\eta_{\varepsilon }({\mathbb{Z}}^{n})$ of an *n*-dimensional integer lattice ${\mathbb{Z}}^{n}$ and the smoothing parameter $\eta_{\varepsilon }(ℒ)$ of a general lattice ℒ.

**Lemma 16.** *If $ℒ\subseteq {\mathbb{R}}^{n}$ is n-dimensional full-rank lattice, then*


$$\eta_{\varepsilon }(ℒ)\leq \tilde{bl}(ℒ)\eta_{\varepsilon }({\mathbb{Z}}^{n}).$$

*Proof*: Assume ℒ=ℒ(𝐁), where $𝐁=\{{𝐛}_{1},{𝐛}_{2},...,{𝐛}_{n}\}$, and


$$\tilde{bl}(ℒ)=\|\tilde{𝐁}\|=\max_{1\leq i\leq n}\|{\tilde{𝐛}}_{i}\|.$$

Let 𝐱∈ℒ. By the definition of a lattice, there exist integers $x_{1},x_{2},...,x_{n}$, such that $𝐱=x_{1}{𝐛}_{1}+x_{2}{𝐛}_{2}+...+x_{n}{𝐛}_{n}$. Combining the equation $𝐁=\tilde{𝐁}𝐔$, we obtain


$$𝐱=x_{1}{𝐛}_{1}+x_{2}{𝐛}_{2}+...+x_{n}{𝐛}_{n}$$

$$=x_{1}{\tilde{𝐛}}_{1}+x_{2}(u_{1,2}{\tilde{𝐛}}_{1}+{\tilde{𝐛}}_{2})+...+x_{n}(u_{1,n}{\tilde{𝐛}}_{1}+...+{\tilde{𝐛}}_{n})$$

$$=(x_{1}+u_{1,2}x_{2}+...+u_{1,n}x_{n}){\tilde{𝐛}}_{1}+...+(x_{n-1}+u_{n-1.n}x_{n}){\tilde{𝐛}}_{n-1}+x_{n}{\tilde{𝐛}}_{n}.$$

Let $s_{i}=\frac{s}{\left|{\tilde{𝐛}}_{i}\right|}$, then


$$\rho_{s}(𝐱)=e^{-\frac{\pi }{s^{2}}|𝐱{|}^{2}}$$

$$=e^{-\frac{\pi }{s^{2}}[x_{n}^{2}|{\tilde{𝐛}}_{n}{|}^{2}+...+(x_{1}+u_{1,2}x_{2}+...+u_{1,n}x_{n}{)}^{2}|{\tilde{𝐛}}_{1}{|}^{2}]}$$

$$=e^{-\frac{\pi }{s_{n}^{2}}x_{n}^{2}-...-\frac{\pi }{s_{1}^{2}}(x_{1}+u_{1,2}x_{2}+...+u_{1,n}x_{n}{)}^{2}}$$

$$=\rho_{s_{n}}(x_{n})\rho_{s_{n-1}}(x_{n-1}+u_{n-1,n}x_{n})\cdots \rho_{s_{1}}(x_{1}+u_{1,2}x_{2}+...+u_{1,n}x_{n}).$$

Therefore, according to Lemma 6, we conclude that:


$$\rho_{s}(ℒ)=\sum_{𝐱\in ℒ}\rho_{s}(𝐱)$$

$$=\sum_{x_{1},...,x_{n}\in \mathbb{Z}}\rho_{s_{n}}(x_{n})\rho_{s_{n-1}}(x_{n-1}+u_{n-1,n}x_{n})\cdots \rho_{s_{1}}(x_{1}+u_{1,2}x_{2}+...+u_{1,n}x_{n})$$

$$\leq \sum_{x_{2},...,x_{n}\in \mathbb{Z}}\rho_{s_{n}}(x_{n})\cdots \sum_{x_{1}\in \mathbb{Z}}\rho_{s_{1}}(x_{1})$$

$$=\sum_{x_{2},...,x_{n}\in \mathbb{Z}}\rho_{s_{n}}(x_{n})\cdots \rho_{s_{1}}(\mathbb{Z})$$

$$\leq ...\leq \rho_{s_{n}}(\mathbb{Z})\rho_{s_{n-1}}(\mathbb{Z})\cdots \rho_{s_{1}}(\mathbb{Z}).$$

According to Lemma 5, we have


$$\rho_{1/s}({ℒ}^{⋆})\leq \rho_{1/s_{1}}(\mathbb{Z})\rho_{1/s_{2}}(\mathbb{Z})\cdots \rho_{1/s_{n}}(\mathbb{Z}).$$

Let *i*_0_ be such that $\left|{\tilde{𝐛}}_{i_{0}}|=\tilde{bl}(ℒ\right)$. Then,


$$\rho_{1/s}({ℒ}^{⋆})\leq (\rho_{|{\tilde{𝐛}}_{i_{0}}|/s}(\mathbb{Z}){)}^{n}$$

$$=\prod_{i=1}^{n}\sum_{x_{i}\in \mathbb{Z}}\rho_{|{\tilde{𝐛}}_{i_{0}}|/s}(x_{i})$$

$$=\sum_{x_{1},x_{2},...,x_{n}\in \mathbb{Z}}\rho_{|{\tilde{𝐛}}_{i_{0}}|/s}(x_{1})\rho_{|{\tilde{𝐛}}_{i_{0}}|/s}(x_{2})\cdots \rho_{|{\tilde{𝐛}}_{i_{0}}|/s}(x_{n})$$

$$=\sum_{𝐱=(x_{1},x_{2},...,x_{n})\in {\mathbb{Z}}^{n}}\rho_{|{\tilde{𝐛}}_{i_{0}}|/s}(𝐱)$$

$$=\rho_{|{\tilde{𝐛}}_{i_{0}}|/s}({\mathbb{Z}}^{n}).$$

When $\frac{s}{\left|{\tilde{𝐛}}_{i_{0}}\right|}\geq \eta_{\varepsilon }({\mathbb{Z}}^{n})$, it follows that $\rho_{|{\tilde{𝐛}}_{i_{0}}|/s}({\mathbb{Z}}^{n})\leq 1+\varepsilon$. Therefore, when $s\geq |{\tilde{𝐛}}_{i_{0}}|\cdot \eta_{\varepsilon }({\mathbb{Z}}^{n})=\tilde{bl}(ℒ)\cdot \eta_{\varepsilon }({\mathbb{Z}}^{n})$, we have


$$\rho_{1/s}({ℒ}^{⋆})\leq \rho_{|{\tilde{𝐛}}_{i_{0}}|/s}({\mathbb{Z}}^{n})\leq 1+\varepsilon .$$

Thus, we have $\eta_{\varepsilon }(ℒ)\leq \tilde{bl}(ℒ)\eta_{\varepsilon }({\mathbb{Z}}^{n})$. The proof is complete ◻

**Theorem 17.** *Let $ℒ\subseteq {\mathbb{R}}^{n}$ be a full-rank lattice. When n is sufficiently large such that $\sqrt{\frac{24n+1}{n^{2}-1}}\leq \varepsilon \leq \sqrt{2}-1$ , then*

$$\eta_{\varepsilon }(ℒ)\leq \tilde{bl}(ℒ)\sqrt{\frac{\ln((n-1)(1+\frac{2}{\varepsilon }))}{\pi }}.$$ (8)

*Proof*: According to Lemma 16, it directly follows that


$$\eta_{\varepsilon }(ℒ)\leq \tilde{bl}(ℒ)\eta_{\varepsilon }({\mathbb{Z}}^{n})\leq \tilde{bl}(ℒ)\sqrt{\frac{\ln((n-1)(1+\frac{2}{\varepsilon }))}{\pi }}.$$

◻

## 4 Comparative analysis of smoothness parameter bounds

In this section, we will illustrate, through some numerical comparisons, that the upper bound on the smoothing parameter (Eq (8)) obtained by us is superior to the results in References [8] and [13] (that is, Eqs (3) and (5)).

Theoretically, when ε→0 and n→∞, all three values will approach $\sqrt{\ln(\frac{2n}{\varepsilon })}$. Therefore, the differences among them can be neglected at this time.

In lattice-based cryptographic constructions, the parameter *n* typically takes values within [512,8192] (see [10,23–25]). Since most practical schemes require n≥2048 and to minimize ε (where $\sqrt{\frac{24n+1}{n^{2}-1}}\leq \varepsilon \leq \sqrt{2}-1$), we focus specifically on *n* is equal to 2048, 4096, and 8192. This selection is also practically motivated.

As shown in Table 1 (the numerical results are provided in detail in Supporting Information S1_Table), after removing a common factor $\tilde{bl}(ℒ)$, when ε is set to 0.11, 0.22 and 0.33 respectively, our results outperform those in [13] and [8] in terms of numerical precision. Specifically, compared with [8], our results exhibit a more pronounced ε-sensitivity at fixed values of *n*—as the error ε increases, the performance advantage grows. This implies that our results are more applicable in scenarios where higher errors are tolerable. Although in comparison with [13], our method does not maintain a significant advantage across all combinations of parameters ε and *n* it has achieved a systematic improvement in numerical precision. Based on this, our results can serve as an effective alternative estimator to [13] for the parameter configuration requirements of high-dimensional lattice-based cryptosystems.

**Table 1:** Comparison of smoothing parameter bounds.

n	ε	Ours:	[13]:	[8]:	[13]-Ours	[8]-Ours
		$\sqrt{\frac{\ln((n-1)(1+\frac{2}{\epsilon }))}{\pi }}$	$\sqrt{\frac{\ln(n-1+\frac{2n}{\epsilon })}{\pi }}$	$\sqrt{\frac{\ln(2n(1+\frac{1}{\epsilon }))}{\pi }}$		
2048	0.11	1.834969	1.835009	1.839414	0.000040	0.004445
4096	0.11	1.894156	1.894175	1.898441	0.000019	0.004286
8192	0.11	1.951538	1.951547	1.955688	0.000009	0.004150
2048	0.22	1.778385	1.778424	1.786865	0.000039	0.008480
4096	0.22	1.839393	1.839412	1.847572	0.000019	0.008179
8192	0.22	1.898431	1.898440	1.906346	0.000009	0.007915
2048	0.33	1.746134	1.746172	1.758210	0.000038	0.012076
4096	0.33	1.808230	1.808249	1.819873	0.000018	0.011643
8192	0.33	1.868253	1.868262	1.879514	0.000009	0.011260

In summary, for the common parameter range (n≥2048) in high-dimensional lattice-based cryptographic systems, our method significantly outperforms Reference [8] in terms of numerical precision and achieves systematic improvements compared with Reference [13]. It provides better theoretical support for practical applications.

## 5 Conclusion

The smoothing parameter $\eta_{\varepsilon }(ℒ)$ was proposed by Micciancio and Regev[3] as a technical tool useful for proving reductions between hard problems on lattices. Since then, it has played a central role in the analysis of lattice-based cryptographic constructions. In this paper, we derive new upper bounds for the smoothing parameter on lattices.

We first consider the case of the one-dimensional integer lattice ℤ, and under certain conditions, we present a more refined upper bound for the smoothing parameter of the one-dimensional integer lattice (see Theorem 10). Then, by establishing an inequality relationship between the smoothing parameters of general *n*-dimensional lattices and *n*-dimensional integer lattices, we extend this result to general *n*-dimensional lattices, and obtain an improved upper bound for the smoothing parameter of general lattices (see Theorem 17). Our results improve upon prior results in the regime of parameters relevant for lattice-based cryptography.

There is still much work that can be attempted regarding the estimation of the upper bounds of the smoothing parameter of lattices. For example, in this paper, how to make the value of ε not be restricted by *n* without relaxing the bounds. Secondly, how to obtain a more refined upper bound for the smoothing parameter of general *n*-dimensional lattices through other mathematical methods. Or whether better results can be obtained on some special lattices, such as integer lattice $ℒ\subseteq {\mathbb{Z}}^{n}$.

## Supporting information

S1 Table MATLAB code for calculating the data in Table 1. (PDF)

## References

[1] Peikert C. A decade of lattice cryptography. FNT Theor Comput Sci. 2016;10(4):283–424. doi: 10.1561/0400000074

[2] Regev O. New lattice-based cryptographic constructions. J ACM. 2004;51(6):899–942. doi: 10.1145/1039488.1039490

[3] Micciancio D, Regev O. Worst-case to average-case reductions based on Gaussian measures. SIAM J Comput. 2007;37(1):267–302. doi: 10.1137/s0097539705447360

[4] Banaszczyk W. New bounds in some transference theorems in the geometry of numbers. Mathematische Annalen. 1993;296:625–35.

[5] Banaszczyk W. Inequalities for convex bodies and polar reciprocal lattices in R n. Discrete Comput Geom. 1995;13(2):217–31. doi: 10.1007/bf02574039

[6] Banaszczyk W. Inequalities for convex bodies and polar reciprocal lattices in R n II: application of K-convexity. Discrete Comput Geom. 1996;16(3):305–11. doi: 10.1007/bf02711514

[7] Regev O. On lattices, learning with errors, random linear codes, and cryptography. J ACM. 2009;56(6):1–40. doi: 10.1145/1568318.1568324

[8] Gentry C, Peikert C, Vaikuntanathan V. Trapdoors for hard lattices and new cryptographic constructions. In: Proceedings of the Fortieth Annual ACM Symposium on Theory of Computing. 2008. p. 197–206. 10.1145/1374376.13744

[9] Gentry C. Toward basing fully homomorphic encryption on worst-case hardness. Lecture Notes in Computer Science. Berlin, Heidelberg: Springer; 2010. p. 116–37. 10.1007/978-3-642-14623-7_7

[10] Espitau T, Wallet A, Yu Y. On gaussian sampling, smoothing parameter and application to signatures. In: International Conference on the Theory and Application of Cryptology and Information Security. Singapore: Springer; 2023. p. 65–97. 10.1007/978-981-99-8739-9_3

[11] Zheng Z, Liu F, Tian K. Modern cryptography volume 2– A classical introduction to informational and mathematical principle. Singapore: Springer; 2023.

[12] Peikert C. Limits on the Hardness of Lattice Problems in ℓ p Norms. Comput Complex. 2008;17(2):300–51. doi: 10.1007/s00037-008-0251-3

[13] Zheng Z, Zhao C, Xu G. Discrete Gaussian measures and new bounds of the smoothing parameter for lattices. AAECC. 2020;32(5):637–50. doi: 10.1007/s00200-020-00417-z

[14] Wei W, Tian C, Wang X. New transference theorems on lattices possessing nϵ-unique shortest vectors. Discrete Math. 2014;315–316:144–55. doi: 10.1016/j.disc.2013.10.020

[15] Chung K-M, Dadush D, Liu F-H, Peikert C. On the lattice smoothing parameter problem. In: 2013 IEEE Conference on Computational Complexity. 2013. p. 230–41. 10.1109/ccc.2013.31

[16] Kirshanova E, Nguyen H, Stehlé D, Wallet A. On the smoothing parameter and last minimum of random orthogonal lattices. Des Codes Cryptogr. 2020;88(5):931–50. doi: 10.1007/s10623-020-00719-w

[17] Zheng Z, Liu F, Lu Y, et al. Cyclic lattices, ideal lattices, and bounds for the smoothing parameter. International forum on financial mathematics and financial technology. Singapore: Springer; 2021. p. 129–53.

[18] Peikert C. An efficient and parallel Gaussian sampler for lattices. Lecture Notes in Computer Science. Berlin, Heidelberg: Springer; 2010. p. 80–97. 10.1007/978-3-642-14623-7_5

[19] Micciancio D, Walter M. Gaussian sampling over the integers: efficient, generic, constant-time. Lecture Notes in Computer Science. Springer; 2017. p. 455–85. 10.1007/978-3-319-63715-0_16

[20] Pöppelmann T, Ducas L, Güneysu T. Enhanced lattice-based signatures on reconfigurable hardware. Lecture Notes in Computer Science. Berlin, Heidelberg: Springer; 2014. p. 353–70. 10.1007/978-3-662-44709-3_20

[21] Zheng Z, Wang X, Xu G, Zhao C. Error estimation of practical convolution discrete Gaussian sampling with rejection sampling. Sci China Inf Sci. 2021;64(3):139104.doi: 10.1007/s11432-019-9919-7

[22] Bennett H, Ganju A, Peetathawatchai P. Just how hard are rotations of Zn? Algorithms and cryptography with the simplest lattice. In: Annual International Conference on the Theory and Applications of Cryptographic Techniques, 2023. p. 252–81. 10.1007/978-3-031-30589-4_9

[23] Espitau T, Fouque PA, Rossi M, et al. MITAKA: a simpler, parallelizable, maskable variant of FALCON. Annual International Conference on the Theory and Applications of Cryptographic Techniques. Cham: Springer; 2022: 222–53. 10.1007/978-3-031-07082-2_9

[24] Ducas L, Postlethwaite EW, Pulles LN, et al. Hawk: module LIP makes lattice signatures fast, compact and simple. International Conference on the Theory and Application of Cryptology and Information Security. Cham: Springer; 2022. p. 65–94. 10.1007/978-3-031-22972-5_3

[25] Espitau T, Niot G, Sun C. Square unstructured integer euclidean lattice signature. In: Submission to the NIST’s post-quantum cryptography standardization process. 2023. https://csrc.nist.gov/csrc/media/Projects/pqc-dig-sig/documents/round-1/spec-files/Squirrels-spec-web.pdf

