# Supplementary material for: New bounds of the smoothing parameter for lattices
Source: PLoS One. 2025 Jul 24;20(7):e0328688. doi: 10.1371/journal.pone.0328688 (PMC12289070; doi:10.1371/journal.pone.0328688)
Supplement: S1 Table — (PDF) [file pone.0328688.s001.pdf]

We have written a MATLAB code for the calculation process in Table 1. We require that the calculation results be rounded to six decimal places. The running results can be referred to the official MATLAB Online (Basic Version), and the website is as follows: https://matlab.mathworks.com/?elqsid=rft76i30k6gdr7ken15r. The following is the MATLAB code:

```
n_values = sym([2048, 4096, 8192]);
epsilon_values = sym([0.11, 0.22, 0.33]);
PI = sym(pi);

bound_ref8      = @(n,e) sqrt( log( 2*n.*(1 + 1./e) ) / PI );
bound_ref13     = @(n,e) sqrt( log( (n-1) + 2*n./e ) / PI );
bound_ours      = @(n,e) sqrt( log( (n-1).*(1 + 2./e) ) / PI );

results = [];
for eps = epsilon_values
    for n = n_values
        ours = bound_ours(n,eps);
        ref13 = bound_ref13(n,eps);
        ref8 = bound_ref8(n,eps);
        diff13 = ref13 - ours;
        diff8 = ref8 - ours;

        results = [results;
            double(n), double(eps), double(ours), double(ref13), double(ref8), double(diff13), double(diff8)];
end
end

fprintf('\\begin{tabular}{|c|c|ccc|cc|}\n')
fprintf('\\hline\n')
fprintf('$n$ & $\\varepsilon$ & \\textbf{Ours} & Ref[13] & Ref[8] & Ref[13]-Ours & Ref[8]-Ours \\\\ \n')
fprintf('\\hline\n')
for i = 1:size(results,1)
    fprintf('%d & %.2f & %.6f & %.6f & %.6f & %.6f & %.6f \\\\ \n',...
            results(i,1), results(i,2),...
            results(i,3), results(i,4), results(i,5),...
            results(i,6), results(i,7));
    if mod(i,3)==0, fprintf('\\hline\n'), end
end
fprintf('\\end{tabular}\n')
```
